# Geometric Analysis and Modeling of Electrospun Nanofiber Mat Deposition in a Top-Down Vertical Configuration

**DOI:** 10.3390/nano16020126

**Published:** 2026-01-18

**Authors:** Margarita Neznakomova, Peter Dineff, Momchil Shopov, Nikolay Nikolov, Dilyana Gospodinova

**Affiliations:** 1 Faculty of Industrial Technology, Technical University of Sofia, 1756 Sofia, Bulgaria; m_neznakomova@abv.bg; 2 Faculty of Electrical Engineering, Technical University of Sofia, 1756 Sofia, Bulgaria; dineff_pd@abv.bg (P.D.); momchil.shopov@gmail.com (M.S.); 3 Faculty of Transport, Technical University of Sofia, 1756 Sofia, Bulgaria; nyky@tu-sofia.bg

**Keywords:** electrospinning, nanofibers, geometric modeling, PVA, three-dimensional reconstruction, process parameters, nanofiber mat geometry, statistical analysis

## Abstract

Electrospinning is a widely used technique for fabricating nanomaterials with tailored morphology and functional properties. This study investigates how two fundamental process parameters—applied voltage and needle tip-to-collector distance—affect the spatial geometry and deposited mass of electrospun nanofiber mats in a top-down vertical electrospinning setup using a 10% (*w*/*v*) PVA solution prepared in deionized water. To support this hypothesis, both experimental measurements and 3D geometric modeling were performed to evaluate the area, perimeter, and deposited mass under different parameter combinations. Digital image analysis and cross-sectional reconstruction were applied to model nanofiber deposition. Regression and ANOVA analyses reveal that the tip-to-collector distance has a statistically significant impact on both area and perimeter of the electrospun nanofiber mat, while the applied voltage in the tested range (15–20 kV) has no significant effect. Interestingly, the total deposited mass shows no clear dependence on either parameter, likely due to startup irregularities or solution droplets.

## 1. Introduction

Over the past two decades, electrospinning has emerged as a prominent nanotechnology method, enabling the production of continuous nanofibers with diameters ranging from tens of nanometers to a few micrometers. Its simplicity, versatility, and cost-effectiveness have driven the development of laboratory and industrial equipment, accompanied by a surge in scientific publications exploring both fundamental and applied aspects of the process [1,2,3,4]. Electrospun nanofibers possess unique properties, such as high surface-to-volume ratio and tunable porosity, making them suitable for a wide range of applications [5,6]. These include water and air filtration membranes, energy storage devices (e.g., supercapacitors, batteries, and solar cells), scaffolds for tissue engineering, wound dressings, drug delivery systems, blood filtration membranes, and sensor applications [1,7,8,9,10,11,12].

**Figure 1:**
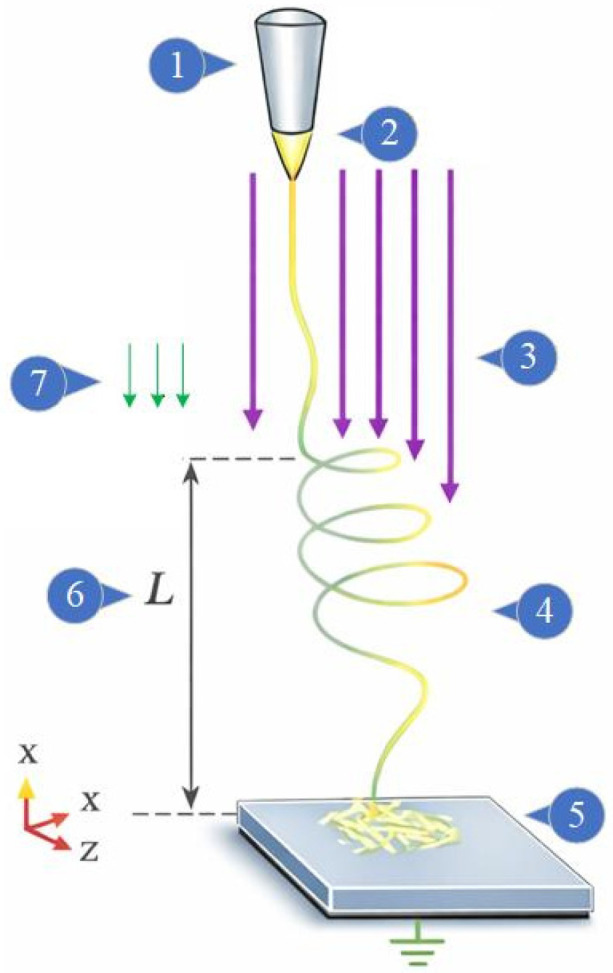
Schematic illustration of the top-down vertical electrospinning process: (1) needle (spinneret), (2) Taylor cone, (3) electric field E, (4) electrospinning jet, (5) grounded collector, (6) needle tip-to-collector distance L, and (7) gravitational acceleration *g*.

Various electrospinning configurations have been developed to meet different application needs. The two most common arrangements are top-down and bottom-up vertical and horizontal setups, differentiated by the orientation of the electric field and the position of the collector relative to the nozzle. The top-down vertical electrospinning configuration offers the advantage of gravity acting in the same direction as the electric field (Figure 1). This enhances jet stability and facilitates uniform fiber collection. According to Suresh et al. [13], to minimize gravity-induced jet sagging and to enhance collection efficiency, the electrospinning setup was operated in a vertical top-down configuration, for which gravity acts in the direction of the electric field. Prior studies report that while gravity is weaker than the electric field, it can still influence the Taylor cone, jet trajectory, and collection rate—effects that are minimized at low flow rates. Researchers such as Caloian et al. have demonstrated the successful production of both two- and three-dimensional nanofiber structures using predefined templates [14], while others, like Zare et al., have explored the fabrication of smart nanomaterials for applications in medicine, sensors, and functional textiles [15].

**Figure 2:**
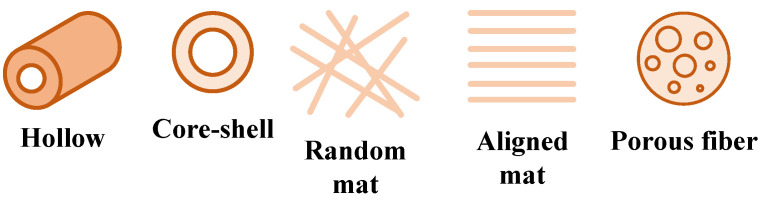
Schematic examples of common nanofiber and nanofiber mat architectures reported in the electrospinning literature. The figure is provided for conceptual illustration only and does not represent results analyzed in the present study.

A key advantage of electrospinning lies in its ability to produce nanofibers with a wide variety of morphologies tailored to specific applications. Structured nanofibers—such as hollow [7,16,17,18,19], mesoporous [7,20,21], core–shell [7,22,23,24], and random or aligned mats [7]—are of particular interest due to their controllable geometry and orientation (Figure 2). However, precise control over the deposition process remains challenging, especially when attempting to tailor the fiber trajectory and stacking behavior on the collector surface [4,25]. The ability to manipulate these properties is critical, as they directly influence the mat’s porosity, mechanical strength, conductivity, and permeability, all of which are crucial for specific application performance [26].

The electrospinning process involves complex electrohydrodynamic phenomena occurring in the presence of a strong electric field (typically 10^5^–10^6^ V.m^−1^), which stretches and accelerates a polymer solution jet toward a grounded collector [4,6,27,28]. The jet initially follows a straight trajectory until, due to bending instabilities as described by Earnshaw’s theorem, it enters a whipping phase, forming a chaotic, spiraling path [3,4,28,29,30]. This dynamic behavior causes further thinning of the fiber and significantly influences deposition distribution [31]. Despite numerous experimental studies, a lack of detailed investigation remains, particularly concerning the influence of ambient airflow and environmental factors on fiber path stability [32,33]. The geometry and construction of the electrospinning chamber also play an important role, as variations in enclosure shape, airflow confinement, and electrode placement can strongly affect jet stability and overall deposition behavior. The dependence on multiple interacting factors complicates experimental control and reduces repeatability. As such, there is a growing need for advanced numerical simulations that can capture the full three-dimensional nature of the jet and deposition behavior.

In this context, numerical modeling serves as a powerful tool to complement experimental work by providing insight into the complex interactions governing fiber behavior. Over recent years, electrospinning modeling has advanced significantly, encompassing a wide range of approaches aimed at describing jet dynamics, electric field distributions, and deposition behavior [33]. Early modeling efforts focused on reduced-order descriptions of charged jet stability and bending or whipping instabilities, providing fundamental insight into jet elongation mechanisms and trajectory evolution [33,34]. More recent studies have incorporated multiphysics numerical frameworks, combining electrostatic forces, fluid dynamics, air drag, and solvent evaporation to better reproduce experimentally observed jet paths and deposition patterns [33]. In parallel, modeling of the electric field and spinneret–collector geometry has been shown to play a critical role in understanding deposition footprint, collector coverage, and scale-up configurations, including multi-nozzle and modified collector designs [33,35]. Additionally, data-driven and regression-based models have emerged as efficient alternatives for capturing process–structure relationships and enabling process control when full multiphysics simulations are computationally prohibitive [36]. Within this context, there remains a need for compact, geometry-oriented modeling approaches that directly link processing parameters to macroscopic deposition characteristics under experimentally relevant configurations.

Electrospinning outcomes are known to be influenced by a wide range of processing, material, and environmental factors. Beyond the applied voltage and the needle tip-to-collector distance, electric field homogenization—particularly in multi-nozzle and modified collector configurations—has been shown to strongly affect jet stability and deposition uniformity [37]. Solution properties such as polymer concentration and electrical conductivity also play a critical role in determining jet stretching, fiber formation, and deposition behavior [38]. In addition, ambient conditions, including temperature and relative humidity, as well as external stimuli such as light exposure, have been reported to significantly influence electrospinning dynamics and nanofiber deposition [39].

In the present study, the applied voltage and the needle tip-to-collector distance were deliberately selected as the primary variables, as they represent the most fundamental and directly controllable parameters governing jet initiation, acceleration, and flight length in a top-down vertical electrospinning configuration. Other factors were kept constant in order to isolate their individual effects on macroscopic deposition geometry and to enable a clear statistical and geometric analysis.

The aim of this study is to investigate the influence of two key process parameters—the applied high DC voltage and the needle tip-to-collector distance—on the spatial geometry and mass distribution of electrospun mats with nanoscale fibers. Building upon previous studies by Suresh et al. [13], Caloian et al. [14], and Zare et al. [15], this work evaluates these effects through a combination of experimental measurements and three-dimensional geometric modeling in a top-down vertical electrospinning configuration.

The outcomes provide new understanding of nanofiber mat morphology under controlled variations of key parameters. The top-down vertical configuration considered here has received limited attention in previous modeling studies; thus, the present work contributes a spatially resolved perspective on nanofiber deposition under practical electrospinning conditions.

It is important to note that the present work focuses on macroscopic geometric descriptors of nanofiber deposition rather than fiber-level morphology. Accordingly, microstructural analysis, such as SEM imaging, falls outside the scope of this study and is identified as a direction for future investigation.

During electrospinning, a polymer jet is ejected from the nozzle and initially travels in a straight line due to surface tension and viscoelastic forces, which stabilize its path. As the solvent evaporates and the jet stretches, its diameter decreases and surface charge density increases, leading to electrostatic repulsion and deformation of the droplet, as described by Reneker and Yarin [40,41].

When the jet’s acceleration becomes too low, bending instabilities occur—long-wave perturbations caused by radial electrostatic forces acting on the jet. These instabilities, first detailed by Reneker and Yarin [41], are amplified by the movement of charges along the jet and cause further elongation and thinning of the fibers. According to SalehHudin et al. [42], the fiber stretching increases significantly in the curved segments, facilitating the formation of nanofibers. Typically, three successive bending instabilities are observed before solidification: an initial straight segment, followed by a first bending loop, then a second, smaller coil, and finally a third, even finer loop, as outlined by Reneker and Yarin [41]. These loops play a crucial role in reducing fiber diameter and producing uniform nanofibers.

Electrospinning and electrospraying are distinct electrohydrodynamic (EHD) processes. Since the present study does not include dedicated diagnostics for regime identification (electrospinning vs. electrospraying), we do not attempt strict regime classification. Instead, we focus on an outcome-based macroscopic analysis of the collected deposits produced under a controlled set of EHD processing conditions commonly reported for PVA fiber-mat fabrication, quantifying the geometric characteristics of the deposited mats and evaluating them statistically.

The present study does not aim to resolve the solvent evaporation state or to distinguish between closely related electrohydrodynamic regimes during jet flight. Instead, the analysis focuses on the resulting macroscopic deposition geometry under processing conditions commonly associated with electrospinning of PVA solutions.

To address this hypothesis, the remainder of the paper is organized as follows. Section 2 describes the materials and experimental methodology. Section 3 presents and analyzes the experimental results. Section 4 discusses the key findings, and Section 5 concludes the study and outlines directions for future work.

The present study has three specific objectives:

(i) to quantify the influence of the applied voltage *U* and needle tip-to-collector distance (L) on geometric descriptors of nanofiber deposition, including mat area and perimeter, under top-down vertical electrospinning conditions;
(ii) to evaluate the statistical significance of these parameters using ANOVA and regression analysis, thereby identifying the dominant operating factors governing deposition geometry; and
(iii) to develop a geometric reconstruction methodology that visualizes the spatial expansion of electrospun nanofiber mats based on experimentally extracted boundary contours.

These objectives focus exclusively on macroscopic geometric deposition characteristics rather than fiber-level morphology, which is beyond the scope of the present investigation.

## 2. Experimental Setup and Methodology

### 2.1. Materials and Electrospinning Parameters

It is well established in the literature that the processes of nanoscale fiber formation and production via electrospinning depend on processing parameters [4,11,42,43,44,45], including the applied voltage, the flow rate of the electrospinning solution, and the distance between the needle tip and the grounded collector. The adopted configuration of the electrospinning system, shown in Figure 1, is of the top-down vertical type. The applied voltage is a critical factor, as it influences the amount of charge carried by the polymer solution jet. Additionally, the applied DC voltage determines the repulsive forces between charges as well as the interaction forces between the jet and the external electric field. A high-accuracy variable DC power supply (model: PS/ER30P03, Glassman High Voltage, Inc., High Bridge, NJ, USA) was used to apply voltages in the range of 15–20 kV between the needle tip and the grounded collector. The output high DC voltage was monitored continuously using a built-in digital display. The power supply system operated at a constant voltage value. Three levels of high DC voltage were applied: 15, 17.5, and 20 kV, selected based on prior art and literary guidelines, [12,46,47]. Higher applied voltage is generally associated with thinner polymer fibers, as reported in previous studies.

The needle tip-to-collector distance was selected based on its known influence on solution jet stability. Distances that are too short may not allow sufficient time for jet solidification before deposition, whereas excessively long distances may reduce the effective electric field strength and hinder stable jet attraction to the collector [48,49,50,51,52]. To meet the objectives of this study and generate a statistically relevant dataset for mathematical modeling, the electrospinning process was investigated at six different distances: 10, 12, 14, 16, 18, and 20 cm. Each experimental condition (i.e., high DC voltage value and distance combination) was repeated three times (n = 3) under identical parameters to ensure reproducibility. The presented results are the average values obtained from these independent runs. Standard deviations and variances were calculated and used in subsequent ANOVA and regression analyses.

During all experiments, a constant solution flow rate of D = 1 mL.h^−1^ was maintained using a medical infusion pump (KD Scientific, model: LEGATO 200, Holliston, MA, USA).

The flow rate was kept fixed intentionally, as the purpose of this study was to isolate the effects of voltage and tip-to-collector distance. Variations in flow rate are known to strongly influence jet stability and fiber morphology, and changing this parameter would introduce an additional source of variability that could obscure the geometric trends under investigation.

**Figure 3:**
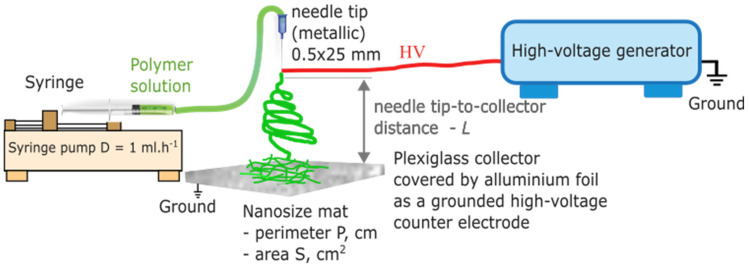
Experimental setup for the top-down vertical electrospinning process. HV denotes the high-voltage (HV) connection applied between the needle and the grounded collector.

The deposition time was kept constant at t = 5 min to ensure an adequate amount of mat was collected on the collector. A single stainless-steel medical needle was used throughout all experiments to ensure consistency (Figure 3). The collector was made of plexiglass and covered with aluminum foil. All electrospinning experiments were conducted under ambient laboratory conditions, with a temperature of 24 ± 1 °C and relative humidity of 40–45%. These environmental factors were monitored using a digital thermohygrometer and remained stable throughout all trials. The outer diameter of the spinneret used was d_out_ = 0.5 mm (25G). The polymer solution for electrospinning was prepared by dissolving PVA (Mw = 72,000 g.mol^−1^, hydrolyzed to a degree of ≥99%) in deionized water at a concentration of 10% *w*/*v*. The solution was heated and stirred at 80 °C for 2 h, then left to stand for 24 h at room temperature to remove bubbles before electrospinning.

### 2.2. Gravimetric and Image Analysis

The experimental approach adopted in this study involves an evaluation based on gravimetric analysis of the deposited nanofiber mat, depending on the applied voltage and the needle tip-to-collector distance. The collector was manufactured using plexiglass as the structural substrate, ensuring both mechanical stability and ease of fabrication. Its surface was subsequently coated with a standard aluminum foil of 16 μm thickness. The aluminum layer was selected not only for its availability and ease of application but also for providing the required electrical conductivity for the system. The mass of the aluminum foil sheets with dimensions of 15 × 15 cm used was measured directly using an analytical balance (model: HM-200-EC, AND, A&D Instruments Ltd., Tokyo, Japan), providing a measurement accuracy up to four decimal places. The deposited mat mass was determined operationally by subtracting the mass of the bare aluminum foil from that of the foil after deposition under identical ambient conditions. No attempt was made to distinguish between polymer mass and potential residual solvent, as the deposited mass is used here solely as a relative, secondary metric to assess process consistency. The experimentally measured quantities used for subsequent analysis are summarized in Table 1.

In addition to gravimetric evaluation, digital image processing was employed to quantify the geometry of the deposited nanofiber mats.

After electrospinning, each nanofiber mat was photographed with a digital camera (Canon EOS 700D, Canon Inc., Tokyo, Japan). A reference scale was pre-applied to the collector, enabling subsequent calibration of the images. The boundary contour of the dense deposition area was traced, while scattered fibers outside the contour were disregarded. The contour curves were imported into ImageJ software (version 1.54g), where the scale was adjusted so that 1 cm in the image corresponded to 1 cm in reality, and both the enclosed area and perimeter were calculated. The extracted two-dimensional boundary contours were subsequently used as geometric inputs for the three-dimensional reconstruction procedure described in Section 2.3.

Each experimental condition was repeated three times (n = 3), which is consistent with exploratory electrospinning studies but which represents a limited sample size from a statistical perspective.

### 2.3. Three-Dimensional Geometric Reconstruction of Nanofiber Deposition

The three-dimensional representation of the deposited nanofiber mats was obtained through a purely geometric reconstruction based on experimentally measured two-dimensional boundary contours.

After electrospinning, each deposited mat was photographed under controlled conditions using a fixed camera position. A reference scale was applied to enable accurate spatial calibration. The images were processed in ImageJ software (version 1.54g; NIH, USA), where the boundary of the dense nanofiber deposition zone was manually traced. Scattered fibers outside this zone were excluded to ensure consistency.

**Figure 4:**
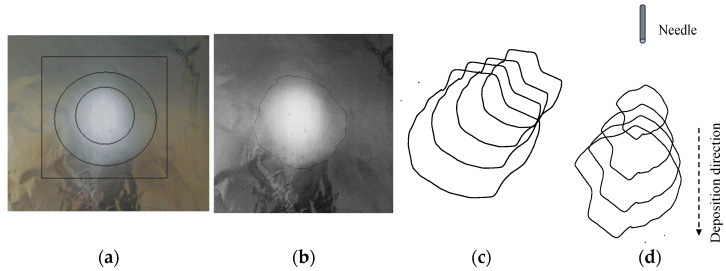
Schematic overview of the procedure for three-dimensional geometric reconstruction of nanofiber mat deposition: (**a**) digital image of an electrospun nanofiber mat deposited on an aluminum collector; (**b**) manually extracted boundary contour of the dense nanofiber deposition region obtained using ImageJ; (**c**) spatial arrangement of the extracted two-dimensional boundary contours according to the top-down electrospinning configuration; (**d**) resulting three-dimensional geometric visualization.

Figure 4 illustrates the methodological workflow and does not represent a physical simulation of the electrospinning process.

For each experimental condition, closed contour curves corresponding to different needle tip-to-collector distances were extracted. The software was used to calculate the enclosed area and perimeter of each contour. These two-dimensional contours were then exported and arranged along the deposition axis according to the actual spatial configuration of the electrospinning setup.

Geometric transformations, including translation, rotation, and inclination, were applied to reflect the relative spatial positions of the contours in the top-down vertical electrospinning configuration. Intermediate interpolated contours were generated using ImageJ shape interpolation tools to visually approximate continuity between successive sections.

The resulting three-dimensional model represents a qualitative spatial reconstruction of nanofiber mat expansion and is intended solely to illustrate deposition geometry for comparative analysis between different process parameters.

**Figure 5:**
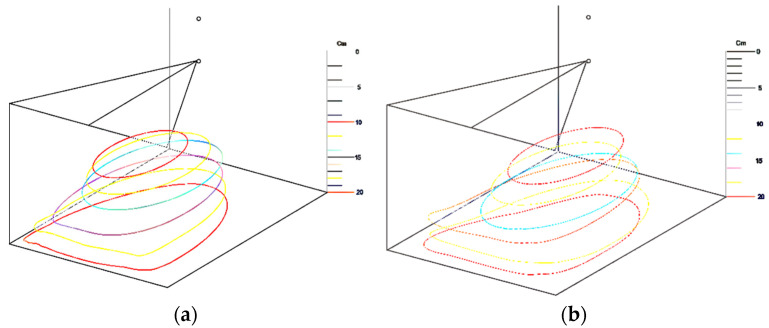
Spatial arrangement of the experimentally extracted boundary contours corresponding to different electrospinning conditions (applied voltages of 15 kV (**a**) and 20 kV (**b**) using a 25G needle). The contours represent the outlines of the dense nanofiber deposition zone and serve as input for the subsequent geometric reconstruction.

The extracted boundary contours for all experimental conditions were then arranged along the deposition axis according to the physical configuration of the top-down setup. The resulting spatial arrangement of the experimental contours is shown in Figure 5.

**Figure 6:**
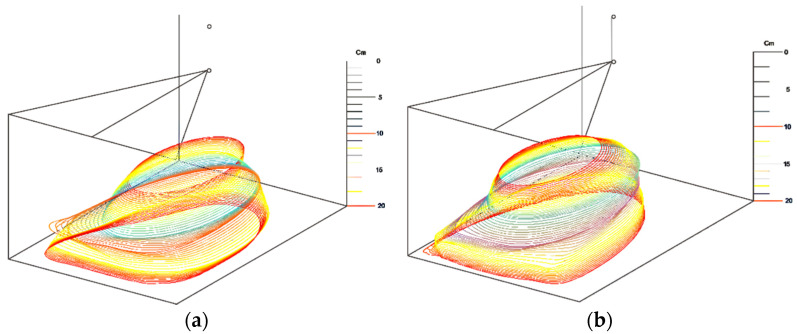
Qualitative three-dimensional geometric reconstruction of nanofiber mat expansion for the electrospinning conditions tested using a 25G needle: U = 15 kV (**a**) and U = 20 kV (**b**). The visualization was generated by arranging the experimentally extracted boundary contours and inserting ten interpolated shapes between successive contours using the ImageJ Shape Interpolation tool. The interpolated shapes enable smooth geometric transitions and do not represent physical cross-sections.

To enable qualitative three-dimensional visualization, intermediate geometric shapes were inserted between successive experimental contours. Ten interpolated shapes were generated using the ImageJ Shape Interpolation tool. These interpolated shapes do not represent physical cross-sections; instead, they provide smooth geometric transitions between adjacent contours, allowing the construction of a continuous 3D envelope. The resulting three-dimensional visualization is presented in Figure 6.

This reconstruction is intended solely as a geometric visualization to illustrate how the deposition zone expands under different process conditions. It is not a physics-based simulation and does not account for jet dynamics, fiber–fiber interactions, or electrohydrodynamic effects.

## 3. Results

### 3.1. Geometric Analysis of the Deposited Nanofiber Mats

The geometric characteristics of the deposited nanofiber mats were evaluated using the experimentally extracted boundary contours described in Section 2.3. For each electrospinning condition, the corresponding contour was used to determine two macroscopic descriptors of the deposition zone: the enclosed area (S) and the perimeter (P). These quantities provide insight into the spatial expansion and boundary complexity of the nanofiber mats formed under different applied voltages and tip-to-collector distances.

The experimentally measured quantities and derived geometric parameters used for the subsequent analysis are summarized in Table 1.

**Figure 7:**
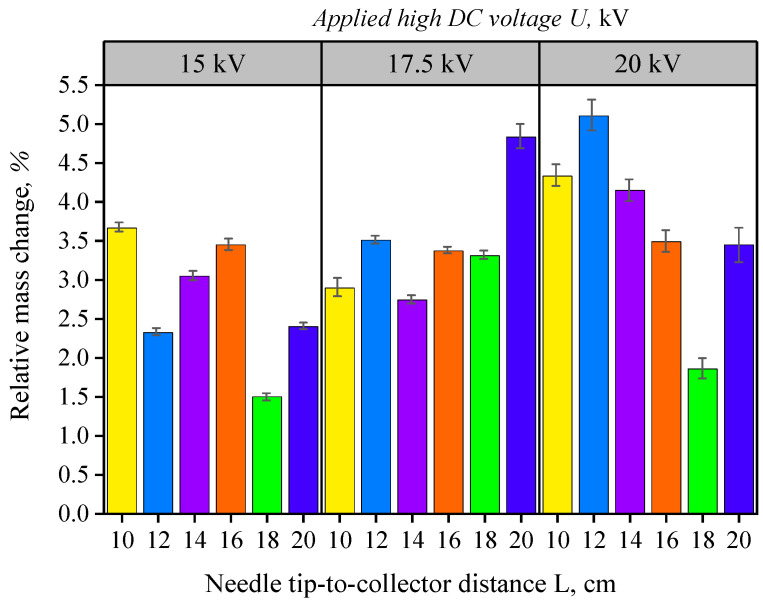
Diagram of the electrospun mat’s mass change as a function of needle tip-to-collector distance using a base 10% PVA solution at a high DC voltage value of U = 15 kV, 17.5 kV, and 20 kV, with a blunt-tipped 25G medical needle and an electrospun solution flow rate of D = 1 mL/h. Bar colors correspond to the investigated needle tip-to-collector distances (L = 10, 12, 14, 16, 18, and 20 cm). Error bars represent the standard deviation calculated from three independent experiments (n = 3).

To evaluate the mass of the deposited mat, which serves as an indicator of the electrospinning process itself, an analysis was conducted to track the change in sample mass resulting from mat deposition (Figure 7). The gravimetric analysis reveals a clear trend of increased sample mass following the electrospinning process. This trend is most pronounced at an applied voltage of 20 kV.

The diagram in Figure 7 illustrates the variability of M_MAT_ across conditions and confirms the absence of a systematic trend with respect to either *U* or *L*. This behavior can be attributed to the transient nature of the initial electrospinning stage, during which both nanofibers and residual solution droplets may be deposited on the collector. This effect introduces additional variability in gravimetric measurements, particularly at short deposition times.

It should be noted that gravimetric measurements provide an integral assessment of deposited material but do not distinguish between fibers and residual droplets formed during the initial stages of electrospinning. Complementary microstructural characterization (e.g., detailed fiber-level morphology assessment) is beyond the scope of the present work and will be considered in future studies.

Within the examined range of operating parameters, the greatest deposited mass was observed at an applied voltage of 20 kV and a tip-to-collector distance of 12 cm. This local maximum should be interpreted as indicative rather than definitive, due to the variability inherent in gravimetric measurements and the limited number of repetitions.

The area of the deposited nanofiber mat is a critical parameter, as it determines the suitability of the material for its intended application. In some cases, a smaller area with less deposited mat is sufficient, while in others, a larger area may be required.

**Figure 8:**
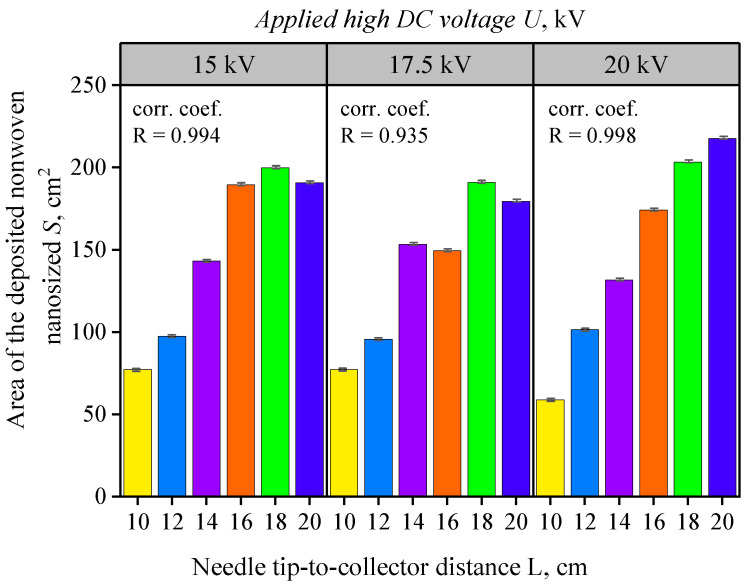
Diagram of the deposited mat area *S* as a function of the applied high DC voltage U = 15, 17.5, and 20 kV and the needle tip-to-collector distance *L*, using a blunt-tipped 25G medical needle and a solution flow rate of D = 1 mL/h. Bar colors correspond to the investigated needle tip-to-collector distances (L = 10, 12, 14, 16, 18, and 20 cm). The “corr. coef.” value (R) denotes the Pearson correlation coefficient between L and S for each voltage. Error bars represent mean ± SD (n = 3).

The diagram in Figure 8, which illustrates the area covered by the mat as a function of the applied voltage and tip-to-collector distance, reveals several clear trends:

(i) For all three tested voltage values (U = 15, 17.5, and 20 kV), the mat-covered area increases as the tip-to-collector distance increases;
(ii) This relationship appears linear, with a sufficiently high correlation coefficient (R > 0.935), indicating a strong linear dependence between the tip-to-collector distance and the electrospun area.

**Figure 9:**
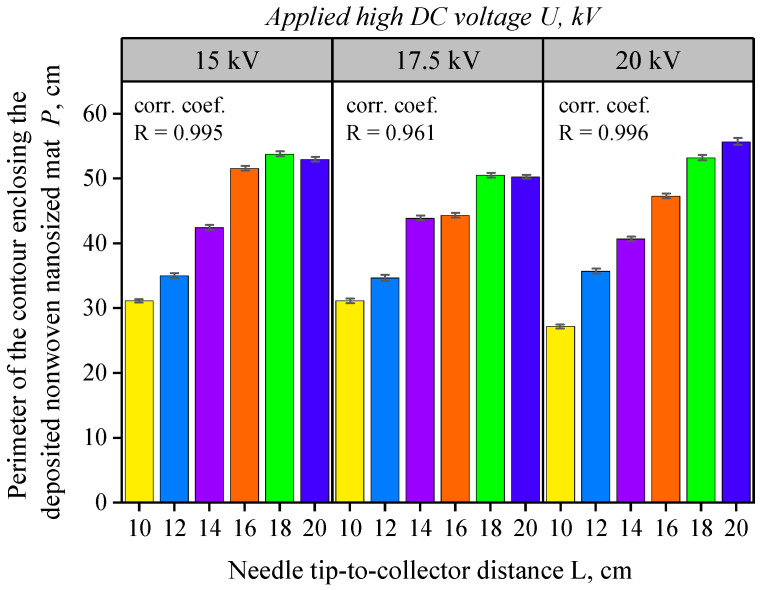
Column diagram of the nanofiber mat perimeter *P* (cm) as a function of the collector distance, at three applied voltage values U = 15, 17.5, and 20 kV, using a 25G nozzle and a solution flow rate of D = 1 mL/h. Bar colors correspond to the investigated tip-to-collector distances (L = 10, 12, 14, 16, 18, and 20 cm). The “corr. coef.” value (R) denotes the Pearson correlation coefficient between L and P for each voltage. Error bars represent mean ± SD (n = 3).

Figure 9 provides interesting insights regarding the perimeter enclosing the deposited nanofiber mat. The isometric values show that the perimeter remains approximately equal across the three different applied voltages, U. Furthermore, for all three voltage levels studied, the maximum perimeter of approximately 52 cm was observed at tip-to-collector distances between 18 and 20 cm. Again, a high correlation coefficient (R > 0.96) confirms a linear relationship between the perimeter P of the deposited mat and the tip-to-collector distance L.

### 3.2. Mathematical and Statistical Analysis

To quantitatively assess the influence of applied voltage U and needle tip-to-collector distance L on the geometry of the electrospun mat, a multiple linear regression model was constructed using the following general form (Equation (1)):

(1) $$Y=b_{0}+b_{1}U+b_{2}L+b_{3}\left(U\cdot L\right)+\varepsilon ,$$

where Y is the dependent variable (either nanofiber mat area S, perimeter P, or mass M_MAT_), U is the applied voltage in kV, L is the tip-to-collector distance in cm, b_0_, b_1_, b_2_ and b_3_ are regression coefficients, and ε represents the random error.

The regression analysis was performed using a standard least-squares fitting approach. To determine the statistical significance of each model term, Analysis of Variance (ANOVA) was conducted. A significance level of α = 0.05 was adopted.

For the response variable S (nanofiber mat area), the ANOVA results indicated that the tip-to-collector distance L has a statistically significant effect (*p* < 0.01), while the applied voltage U and the interaction term U × L were not significant (*p* > 0.05). Similar trends were observed for P (perimeter). No statistically significant effect was found on M_MAT_ (mass). Table 2 summarizes the regression coefficients and corresponding *p*-values for each factor and interaction term across the three response variables.

The analysis indicates that the needle tip-to-collector distance (*L*) has a statistically significant effect on both the mat area (*S*) and perimeter (*P*), whereas the applied voltage (*U*) and the interaction term (*U* × *L*) do not exhibit statistically significant effects at the 95% confidence level. The positive coefficient associated with *L* indicates that increasing the working distance leads to an expansion of the deposited mat area and perimeter. This behavior can be attributed to enhanced jet elongation and whipping instabilities prior to deposition, as longer flight times allow increased stretching of the polymer jet.

**Figure 10:**
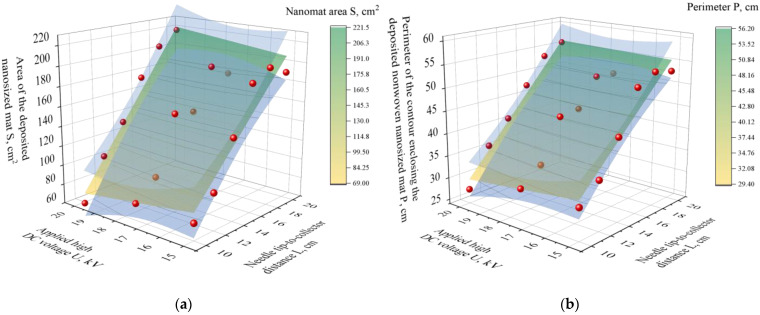
Three-dimensional regression surfaces for nanofiber mat area *S* (**a**) and perimeter of the contour enclosing the deposited mat P (**b**) versus applied high DC voltage *U* and needle tip-to-collector distance *L*, with 95% confidence bounds (blue) and measured data points (red), yellow–green surface: model prediction.

The absence of statistical significance for *U* within the investigated range suggests that voltages between 15 and 20 kV were sufficient to initiate stable fiber formation but did not substantially influence the macroscopic geometric characteristics of the deposited mats. These findings are consistent with established electrospinning mechanisms reported in the literature, which highlight the tip-to-collector distance as a key parameter governing jet travel time, solvent evaporation, and the transition between stable and whipping regimes [3]. To visually illustrate these relationships, contour plots were generated for the deposited mass (*M_MAT_*) and mat area (*S*) (Figure 10a,b). In agreement with the ANOVA results, the contour plot for *M_MAT_* does not reveal a clear dependence on either processing parameter, whereas the plot for *S* shows a systematic increase with increasing *L*, confirming that the tip-to-collector distance is the dominant factor controlling deposition geometry under the investigated conditions.

Statistical analyses, including ANOVA and regression modeling, confirm that the needle tip-to-collector distance exerts a dominant influence on both the area and perimeter of the electrospun mats (*p* < 0.01), while the applied voltage does not exhibit significant effect within the tested range (15–20 kV). These findings support the initial hypothesis and indicate that spatial deposition control can be achieved primarily through adjusting collector distance.

## 4. Discussion

Within the moderate voltage interval investigated in this study (15–20 kV), the applied electric field is sufficient to establish a stable and continuous electrospinning jet. Consequently, further increases in voltage within this narrow range exert only a marginal influence on macroscopic deposition geometry. This behavior is consistent with the fact that, once the whipping regime is initiated, jet elongation and spatial dispersion are governed primarily by bending instabilities rather than by incremental changes in the initial electric field strength [3,53].

In contrast, the needle tip-to-collector distance (*L*) exhibits a pronounced influence on nanofiber mat geometry. Increasing *L* extends the jet flight time, allowing more complete jet stretching, whipping, and lateral expansion prior to deposition. As a result, larger deposition areas and increased perimeters are obtained. These observations are consistent with previous studies on vertically oriented electrospinning systems, where the working distance has been identified as a dominant parameter controlling nanofiber spread and deposition footprint [31,49]. The statistical significance of *L* and the comparatively weak influence of *U* are further supported by the regression and ANOVA results summarized in Table 2.

**Figure 11:**
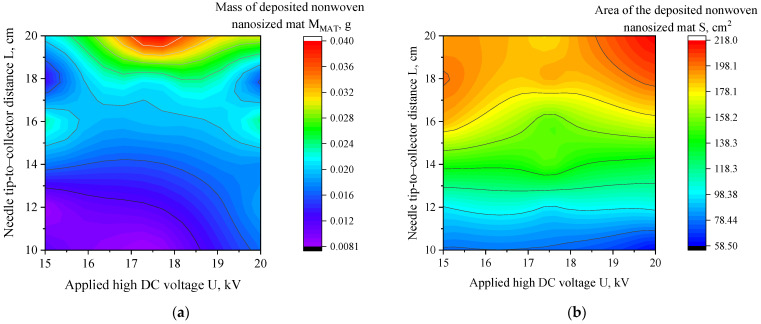
Contour plots showing the dependence of (**a**) deposited mat mass (*M_MAT_*) and (**b**) area (*S*) on the applied high DC voltage (*U*) and needle tip-to-collector distance (*L*). Both parameters show the dominant distance influence, with minimal or no statistically significant influence of high DC voltage value in the tested range.

The response surface and contour plots presented in Figure 11 provide a visual confirmation of these trends. The contour map for deposition area shows a clear and systematic increase with increasing *L*, while variations in *U* produce only minor changes. This agreement between statistical analysis and geometric visualization reinforces the conclusion that the tip-to-collector distance is the primary parameter governing macroscopic deposition geometry under the investigated conditions.

From an application perspective, these findings are directly relevant to practical electrospinning process design. For applications requiring large-area coverage, such as wound dressings or tissue scaffolds, increasing the working distance offers an effective means of controlling mat dimensions without increasing voltage, which may be constrained by safety or equipment limitations. Conversely, applications requiring more compact or higher-density mats, such as sensors or filtration membranes, may benefit from reduced working distances to achieve tighter fiber packing and more localized deposition.

The deposited mass (*M_MAT_*) did not exhibit statistically significant dependence on either the applied voltage or the needle tip-to-collector distance. This outcome is consistent with theoretical expectations for stable electrospinning conditions, under which most of the ejected polymer is collected on the substrate and solvent evaporation proceeds in a comparable manner under controlled ambient conditions. Accordingly, deposited mass is treated here as a secondary, control metric confirming process consistency, while the primary focus of the present study is placed on macroscopic deposition geometry.

While several of the observed trends—such as the dominant influence of the needle tip-to-collector distance on deposition geometry—are qualitatively consistent with long-standing empirical knowledge in electrospinning, the present work provides a quantitative and statistically supported framework that formalizes these observations. By combining controlled experiments, ANOVA-based significance analysis, and three-dimensional geometric reconstruction, this study moves beyond empirical trial-and-error optimization and enables a reproducible assessment of how individual processing parameters govern macroscopic deposition characteristics. Importantly, the proposed approach does not aim to establish a universal predictive law but rather to offer a compact, geometry-oriented modeling methodology applicable within defined operating windows.

It should be emphasized that the results presented in this study are strictly valid under the specific working conditions investigated, including the selected polymer concentration and electrospinning parameters. The conclusions are therefore limited to the experimental window considered. Extension of the applicability of the proposed approach to other polymer systems, solution concentrations, or environmental conditions would require additional dedicated studies and is beyond the scope of the present work.

## 5. Conclusions

This study examined the effect of applied voltage and needle tip-to-collector distance on the geometry and mass of electrospun nanofiber mats using a top-down vertical electrospinning configuration. A combination of experimental measurements, statistical modeling, and surface visualization was employed to characterize the deposited material.

The results demonstrated that the needle tip-to-collector distance is the dominant parameter influencing both the nanofiber mat area and perimeter. In contrast, the applied voltage in the tested range (15–20 kV) showed no statistically significant effect on either geometric or mass parameters. Additionally, the deposited mass did not significantly depend on either variable, suggesting that geometric descriptors may be more sensitive indicators of deposition control.

These findings provide a useful basis for optimizing nanofiber fabrication processes, particularly in applications that require precise control of mat shape and coverage, such as scaffolds for tissue engineering, sensors, and membranes. The study also confirms that top-down vertical electrospinning is a viable alternative to conventional horizontal setups for achieving controlled fiber deposition over large areas.

Future work may extend this methodology to other polymer systems and process parameters, such as flow rate and environmental conditions, to develop a comprehensive predictive model for nanofiber mat formation.

## Figures and Tables

**Table 1:** Experimentally measured quantities and derived geometric parameters used for the statistical and geometric analysis of electrospun nanofiber mats.

Applied Voltage Value U, kV	Needle Tip-To-Collector Distance L, cm	Mass of Deposited Nanofiber Mat M_MAT_, g	Area of the Deposited Nanofiber Mat S, cm^2^	Perimeter of the Contour Enclosing the Deposited Nanofiber Mat P, cm	Mass of Electrospun Nanofiber Mat per Unit Area *ρ_S_* = M_MAT_/S, g.cm^−2^
**15**	10	0.0116	77.12	31.15	1.504
	12	0.0079	97.43	34.00	0.811
	14	0.0173	143.05	42.41	1.209
	16	0.0237	189.47	51.54	1.251
	18	0.0120	199.73	53.76	0.601
	20	0.0210	190.63	52.94	1.102
**17.5**	10	0.0078	77.17	31.15	1.011
	12	0.0119	95.53	34.67	1.246
	14	0.0157	153.20	43.87	1.025
	16	0.0200	149.42	44.31	1.338
	18	0.0203	190.95	50.50	1.063
	20	0.0422	179.39	50.24	2.352
**20**	10	0.0167	58.83	27.19	2.838
	12	0.0188	101.41	35.70	1.854
	14	0.0175	131.67	40.68	1.329
	16	0.0241	174.04	47.26	1.385
	18	0.0147	203.30	53.19	0.723
	20	0.0303	217.61	55.64	1.392

**Table 2:** Regression coefficients and significance (ANOVA results).

Response	Term	Coefficient (b)	*p*-Value	Significance
**S (Area)**	Intercept	11.27	−	−
	Voltage U	−0.12	0.078	Not significant
	Needle tip-to-collector distance L	+0.38	0.003	Significant (✓)
	U × L	−0.02	0.415	Not significant
**P (Perimeter)**	Voltage U	−0.18	0.062	Not significant
	Needle tip-to-collector distance L	+0.44	0.007	Significant (✓)
**M_MAT_ (Mass)**	All terms	−	>0.1	Not significant

## Data Availability

The original contributions presented in this study are included in the article.
